# Strategies for effective implementation and scale-up of a multi-level co-designed men's health initiative “Sheds for Life” in Irish Men's Sheds

**DOI:** 10.3389/frhs.2022.940031

**Published:** 2022-11-03

**Authors:** Aisling McGrath, Noel Richardson, Niamh Murphy

**Affiliations:** ^1^School of Health Sciences, South East Technological University, Waterford, Ireland; ^2^National Centre for Men's Health, South East Technological University, Carlow, Ireland

**Keywords:** Men's Sheds, implementation science, translational research, community-based, men's health, health promotion, participatory research, embedded research

## Abstract

Sheds for Life is a gender-specific tailored men's health initiative engaging “hard-to-reach” men in the Men's Shed setting in Ireland. It is implemented by multiple stakeholders at individual, provider, organization and systems level and thus multiple contextual factors influence its scalability. This research used established implementation science frameworks to guide participatory research approaches that captured the process and identified facilitators of and barriers to implementation and scale-up. Active recruitment, co-design processes, leadership and stakeholder engagement emerged as key facilitators of implementation. Prominent barriers were institutional capacity and funding. Acceptability, adoption and appropriateness of the initiative were high among stakeholders with sustainability largely contingent on funding and staff resources. Findings make a valuable contribution to knowledge by capturing the process involved in the implementation of a complex multi-level men's health intervention. It provides a “how to” guide of strategies to engage hard-to-reach men with health promotion, the operationalization and application of implementation frameworks in community-based health promotion, and the implementation of health promotion in Men's Sheds. Documented barriers and facilitators that impact implementation of a community-based men's health program are rare and provide a valuable blueprint for practitioners, researchers and policy makers in the field.

## Introduction

The burden of ill health in men is caused by multiple complex factors that are particularly exacerbated for vulnerable groups of socially disadvantaged men (1, 2). While it may be perceived that traditional masculine ideals which impede positive men's health behaviors are typical of mainly older men, evidence suggests these barriers remain a systemic issue that continue to pervade through generations (3). Understanding the complexities of masculinities within health systems and how men engage with, and are impacted by them has highlighted a need for tailored men's health programs underpinned by gender-specific approaches (4, 5). This fact is further compounded by the disparity in mortality for men during COVID-19 which was likely a consequence of failure to invest in men's health (6, 7). This need is particularly pertinent for men who are at risk of being more isolated from, or reticent about, accessing formal health services or social supports due to geography, experiences of mental health issues, social disadvantage, unemployment, low educational attainment or significant changes in life course (e.g., retirement)—groups that are considered “hard-to-reach” (HTR) in health endeavors (8). Moreover, designing models of care that are accessible to men and that address changing masculinities across the life course, can be instrumental in reaching out to HTR men while simultaneously acknowledging their diversity (9).

The Men's Sheds (“Sheds”) are autonomous, grassroots organizations that originated in Australia in the 1980s and grew exponentially in Ireland from 2011 following the economic recession. Founded and sustained by Shed members (“Shedders”), membership within Sheds attracts diverse representations of men from different socioeconomic backgrounds, and importantly, are effective in attracting cohorts of HTR men (10–12). The proliferation of Sheds across Ireland was testament to a growing need for men to identify with a space that facilitated meaning, social support, safety and belonging (10, 13). By virtue of their grassroots, member focused approach, Sheds are variable spaces that differ in size, range of activities (e.g., woodwork, music, gardening, art, and mechanics) and resources but have commonality in offering men a safe and familiar environment that fosters a sense of social support and belonging, through developing new skills, shared projects, team work and camaraderie (14, 15). Not surprisingly, Sheds have therefore been identified as inherently health promoting spaces for men (13, 16). Based upon their inherent health promoting qualities and ready access to men who may be reticent to engage with traditional health services, Sheds represent an attractive setting in which to build structured health initiatives. In light of this, Sheds have emerged as an exemplar for the promotion of men's health and wellbeing by health and social policy makers, earmarked as spaces that are capable of engaging HTR men in health endeavors (10, 17). Notwithstanding the fact that Sheds potentially offer a strong foundation upon which to build structured health promotion, tension may arise from imposing formal healthcare upon the informal setting of the Sheds, where its informality is an integral element to its inherent health promotion and where formality may be the very convention men seek to resist (10, 18). Nevertheless, Shed members (Shedders) have demonstrated an appetite for health promotion in Sheds (10), suggesting it is timely to capitalize on this opportunity. The critical consideration in the design, implementation and evaluation of health promotion programs in Sheds is that Shedders are at the center of all decision making and that the ethos of the Shed environment is preserved (10, 18).

Recognizing the utility of Sheds as a means to engage HTR men with health while also understanding the need to prioritize wellbeing for its membership in a tailored and respectful way, the Irish Men's Sheds Association (IMSA) first developed the concept of Sheds for Life (SFL) in 2016 (19). Sheds for Life is a men's health initiative tailored to the Shed setting in Ireland. Through ongoing consultation with stakeholders, Sheds for Life was developed and refined into a 10-week program consisting of four core pillars of a health check, healthy eating, physical activity and mental health along with several option components focusing on life skills and disease prevention (19). A detailed protocol is available which outlines the various components of SFL (19) and the development of this approach will also be further discussed in the context of this research (see Results). Prior to the implementation of a structured SFL program, the IMSA embarked on scoping work at various regional Shed meetings to engage Shedders to identify their health needs and preferences. The IMSA also began to develop partnerships with provider organizations who were actively seeking to engage HTR groups of men in their health promoting initiatives. This resulted in the piloting of discrete wellbeing workshops in Sheds (19). Initial scoping work which sought to investigate how SFL piloting was experienced in practice determined that respecting the Shed environment was critical to the acceptability of SFL and strategic evaluation of the development of SFL would be required to facilitate effective implementation (10). In June 2018 the current authors commenced the formal evaluation of SFL with a dual focus on both efficacy and implementation.

Findings from research show that in order to engage men, particularly those who are HTR, health promotion must include men in decision making and encourage a collaborative process involving all key stakeholders; researchers, practitioners, participants and policy makers (10, 20). Community-based participatory research approaches also emphasize the importance of creating partnerships with the people for whom the research is ultimately meant to benefit (21). Moreover, SFL scoping work highlighted the importance of strengthening ties with local providers and community organizations, an established strategy when seeking to scale-up programs nationally, especially under real world conditions (10, 22). This led to a pragmatic study design using community-based participatory research approaches (CBPR) that were geared toward upholding autonomy and increasing the agency of participants (10). Questions emerged as to the “what” and the “how” of SFL that ought to be evaluated, particularly with regard to reconciling gold standard evaluation methods with the high variability, autonomy and ethos of the Sheds as implementation settings. Moreover, beyond the environment of the Sheds there is also a need to understand the complex intervening variables that act as a backdrop to implementation of SFL (e.g., those at provider, organizational and systems levels) (23, 24). The use of implementation science can be valuable in identifying barriers and facilitators to effectively implementing programs as well as promoting systematic uptake in real world settings from the outset (25). Indeed, implementation science encompasses many of the principles of CBPR, with both approaches linked to improved knowledge translation. These include the engagement of key stakeholders to understand contextual factors, a focus on capacity building, partnership in the research process, and systems development through a cyclical and iterative process with a view to long-term sustainability, (21, 24, 26).

Sheds for Life operates within a complex system of shifting elements such as the diverse and variable contexts of the Sheds and the wider implementation environment, including the competing priorities of provider organizations and systems level funding and polices. As a result, there is a need to continually engage current and emerging stakeholders as well as inform key adaptations and processes that are necessary to implement SFL in multiple locations while executing appropriate implementation strategies to embed SFL in the routine environment of the Shed. Indeed, these dimensions continually evolve over time and require on-going monitoring. Thus, this research was guided by a combination of implementation and evaluation frameworks. While implementation science was used to address implementation issues, there is still a delay when following the traditional route of efficacy-effectiveness-implementation. The speed of moving research findings into routine adoption can be improved by considering hybrid designs that combine elements of effectiveness and implementation research (27, 28). Hybrid designs focus on the dual testing of both effectiveness of the clinical intervention and its implementation. This type of trial design is not dictated by the type of hybrid, meaning that many types of randomized and non-randomized studies can utilize this approach (28). Hybrid type 2 designs are ideal when there is momentum for implementation in terms of system or policy demands (28) - particularly relevant in the case of SFL where there have been calls to implement targeted health promotion in the Sheds supported by a rich landscape of men's health research and policy in Ireland (10).

Alongside the need to identify suitable programs to engage men with health, there is a lack of practical guidance on how to effectively implement and scale-up heath interventions (24). In the context of SFL, scale-up is the deliberate effort to increase impact of SFL so as to benefit more Shedders while fostering more sustainable program development that may influence policy (29). This involves assessing scalability through measuring feasibility, acceptability, costs, sustainability and adaptability (30). The effectiveness-implementation design of this research aimed to engage all key stakeholders in the development, testing, implementation and scale-up of SFL. It aimed to investigate both the process and effectiveness of the SFL intervention with a focus on the key strategies involved in implementation and future scale-up to maximize reach to HTR men within the non-conventional settings of Sheds and the wider implementation environment. A detailed protocol which outlines the effectiveness-implementation design is available (19) as well as work which describes effectiveness outcomes (31). This study discusses the implementation research of SFL in terms of the process of implementation, identification of barriers, facilitators and strategies that impact on implementation outcomes, guided by established implementation frameworks (24, 32, 33). This paper addresses an important gap in the literature by applying an implementation lens to the evaluation of a community-based men's health promotion program using gender-specific approaches. Findings from this research can play a significant role in determining the implementation effectiveness, sustainability, and potential scale-up of the SFL initiative and, more broadly, in terms of the wider rollout of community-based men's health programs.

## Materials and methods

### Research design

A mixed methods process evaluation was used to guide the implementation of SFL guided by a combination of applicable implementation frameworks (24, 32, 33). This consisted of a combination of focus groups, interviews, observations, questionnaires and administrative data (e.g., attendance records). In order to explain or understand implementation outcomes, the perspectives and experiences of a broad representation of stakeholders at the participant, provider, organization and wider systems level were sought. Purposive sampling was used to identify key stakeholders for interview who could inform implementation outcomes across the multi-level implementation environment. Mixed methods were used to inform implementation outcomes. A diverse range of Shed member views were sought from Shed settings based on Shed size (small/large), and geographical location (urban/rural). At the provider, organization and systems level a diverse range of views were sought based on their role within SFL e.g., funder, deliverer, partner, implementer. Semi-structured topic guides and interview schedules were developed for focus groups and interviews. These were designed using a hybrid deductive-inductive approach applying implementation frameworks to assess implementation outcomes, exploring topics areas such as adoption, acceptability, and appropriateness of SFL. These were also designed to allow room for exploring attitudes toward SFL as well as changes in knowledge and behaviors where applicable. The PRACTical planning for Implementation and Scale-up guide (PRACTIS) guide was used as part of an iterative process to characterize parameters of the implementation setting, engage key stakeholders, identity implementation barriers and facilitators, and address potential barriers to implementation within the evolving implementation climate (24). Ongoing consultation with stakeholders was deemed appropriate to the implementation approach as contextual shifts can be unpredictable and assessment of the broader implementation environment required flexibility and iteration (34). The first author was positioned within the organization (IMSA) for the duration of the research which informed ongoing monitoring of the implementation approach. Alongside this, semi-structured interviews (*n* = 19; Provider level *n* = 15, Organizational level *n* = 2, Systems level *n* = 2) were conducted at provider, organizational and systems level using interview schedules which were designed based on the Consolidated Framework for Implementation Research (CFIR) constructs (32) and used to inform a taxonomy of implementation outcomes (33). Implementation monitoring consisted of ongoing engagement with service provider organizations through quarterly stakeholder meetings (*n* = 12). Meetings took place at least twice weekly between the health and wellbeing team responsible for coordinating SFL and the principal researcher from the period of January 2018 to January 2022. Approximately 50 meetings occurred with individual provider organizations and monthly report meetings took place with funding bodies, alongside quarterly financial reports.

The effectiveness evaluation involved following a cohort of SFL participants (*n* = 421) across *n* = 22 Shed settings for up to 12 months to assess impact of SFL on health and wellbeing outcomes. In terms of the assessment of implementation data, this data collection approach was also used to assess outcomes such as cost (35) while administrative data was gathered (Shed numbers and attendance rates) to inform penetration. Throughout this time the first author spent ~500 h among participants within the Sheds setting which facilitated direct observation of SFL in practice as well as observation of Shedders' experiences of SFL. Purposive sampling was also used to conduct focus groups (*n* = 8) with participating Sheds based on Shed size, location and level of attendance in SFL. This approach sought to gather a diverse representation of Shedders' experiences of SFL implementation. Informal short interviews (*n* = 16) were also conducted *ad-hoc* during Shed visits to further inform Shedders' experiences of implementation of SFL. This process was guided by CFIR constructs with a view to also informing the effectiveness of implementation strategies.

### Selection of implementation frameworks

The implementation and sustainment of an effective, evidence-based program in the real-world setting is complex and therefore multiple frameworks are increasingly being used and recommended in studies to address multiple facets of implementation (36–38). The use of theories, frameworks and models, which are often used interchangeably in implementation science can also cause further complexities for researchers (23, 36). Nilsen (23) recommends selecting implementation frameworks based on three overarching aims: (1) describing or guiding the process of translating research into practice (2) understanding the determinants that influence implementation outcomes and (3) evaluating the implementation (23). As the SFL research aimed to evaluate the implementation of the SFL initiative as well as understand the process and determinants of implementation, frameworks that suitably guided the process and evaluation of the research were selected. These frameworks consisted of a determinant framework to specify constructs that may influence the SFL process and predict implementation outcomes, a process framework to specify steps to execute for implementation phases and an evaluation framework to specify multiple levels of outcomes to assess (19).

The process framework applied to SFL implementation was the PRACTical planning for Implementation and Scale-up guide (PRACTIS) (24). The PRACTIS was used in an iterative process to practically guide the implementation process and evaluation in collaboration with key stakeholders. This framework was selected as it incorporated the use of CBPR and is operational in real world contexts, considering the influence of the wider implementation climate (24). In this study, it was used to promote successful implementation and scale-up of SFL. Sheds for Life implementation was guided by four key steps, and will provide structure to the presentation of research findings, namely; characterizing the parameters of the implementation setting; identifying and engaging key stakeholders; identifying implementation barriers and facilitators; and addressing potential barriers to implementation across individual, provider, organizational and systems levels.

The determinant framework used was The Consolidated Framework for Implementation Research (CFIR) (32). This framework was selected to characterize and understand constructs across five domains (intervention characteristics, outer setting, inner setting, characteristics of the individuals involved, and the process of implementation) which interact in complex ways to influence implementation outcomes. The CFIR was used as a practical guide to systematically assess potential barriers and facilitators during SFL implementation as well as guide methods for data collection and analysis. The guide was used in the development of interview schedules as well as in data analysis *via* a deductive approach where key themes were mapped to CFIR constructs across the five CFIR domains (see Table 2).

**Figure 1:**
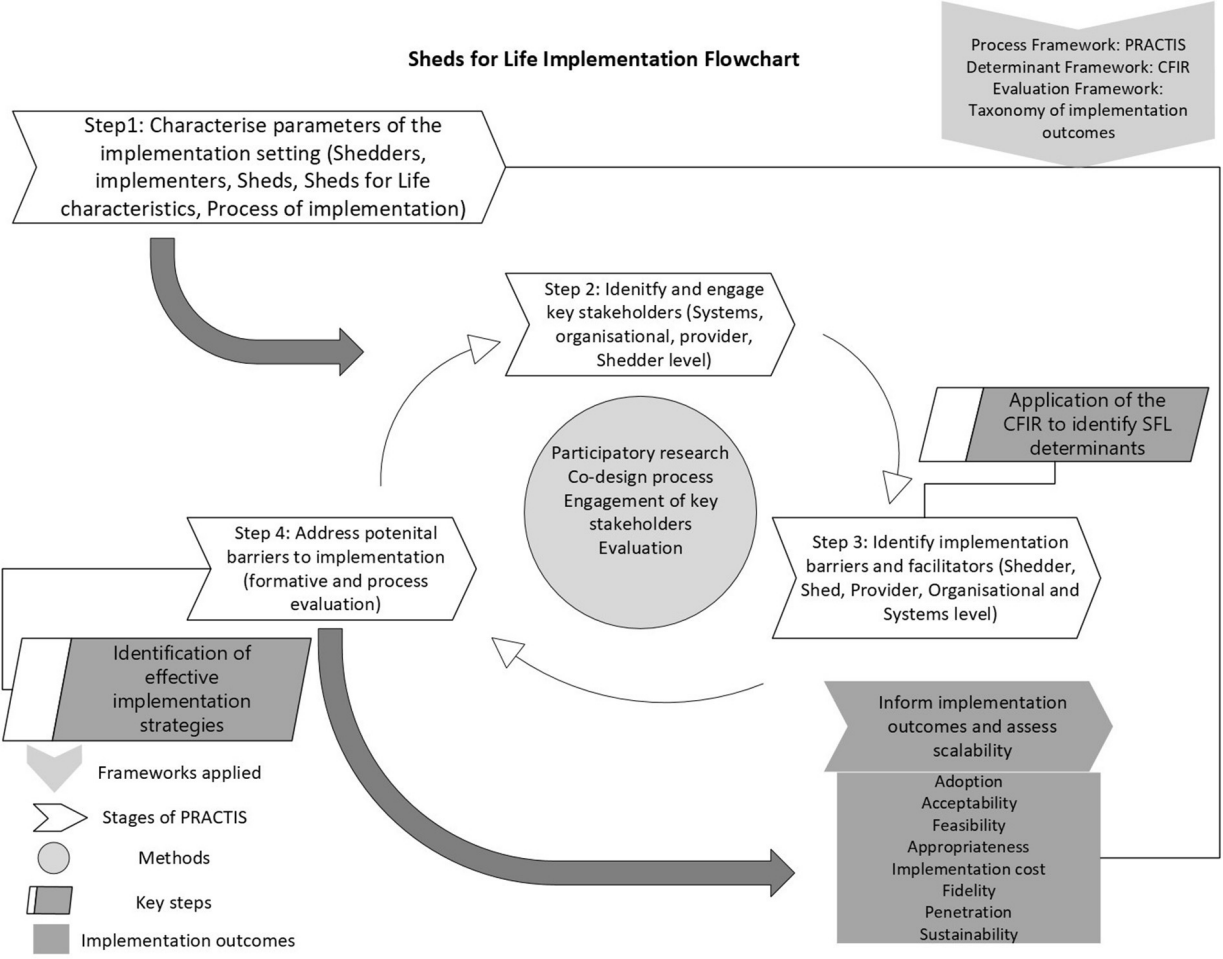
Sheds for Life implementation evaluation flowchart. CFIR, Consolidated Framework for Implementation Research; SFL, Sheds for Life; PRACTIS, Practical planning for Implementation and Scale-up guide; Sheds, Men's Sheds Shedders, Men's Shed members.

The evaluation framework applied to SFL was the taxonomy for implementation outcomes (33). This framework was chosen to inform outcomes pertaining to implementation i.e., acceptability, adoption, appropriateness, feasibility, fidelity, implementations costs, penetration and sustainability. These were assessed in the SFL evaluation using mixed methods to measure implementation effect (see Figure 1). This evaluation framework was selected as the constructs by Proctor et al. (33) have potential to capture participant and provider attitudes (acceptability), behaviors (penetration, adoption) as well as contextual factors (appropriateness, sustainability and implementation cost) (33). Figure 1 depicts the process of SFL implementation and the application of stages of the PRACTIS with use of the CFIR and taxonomy for implementation outcomes.

**Figure 2:**
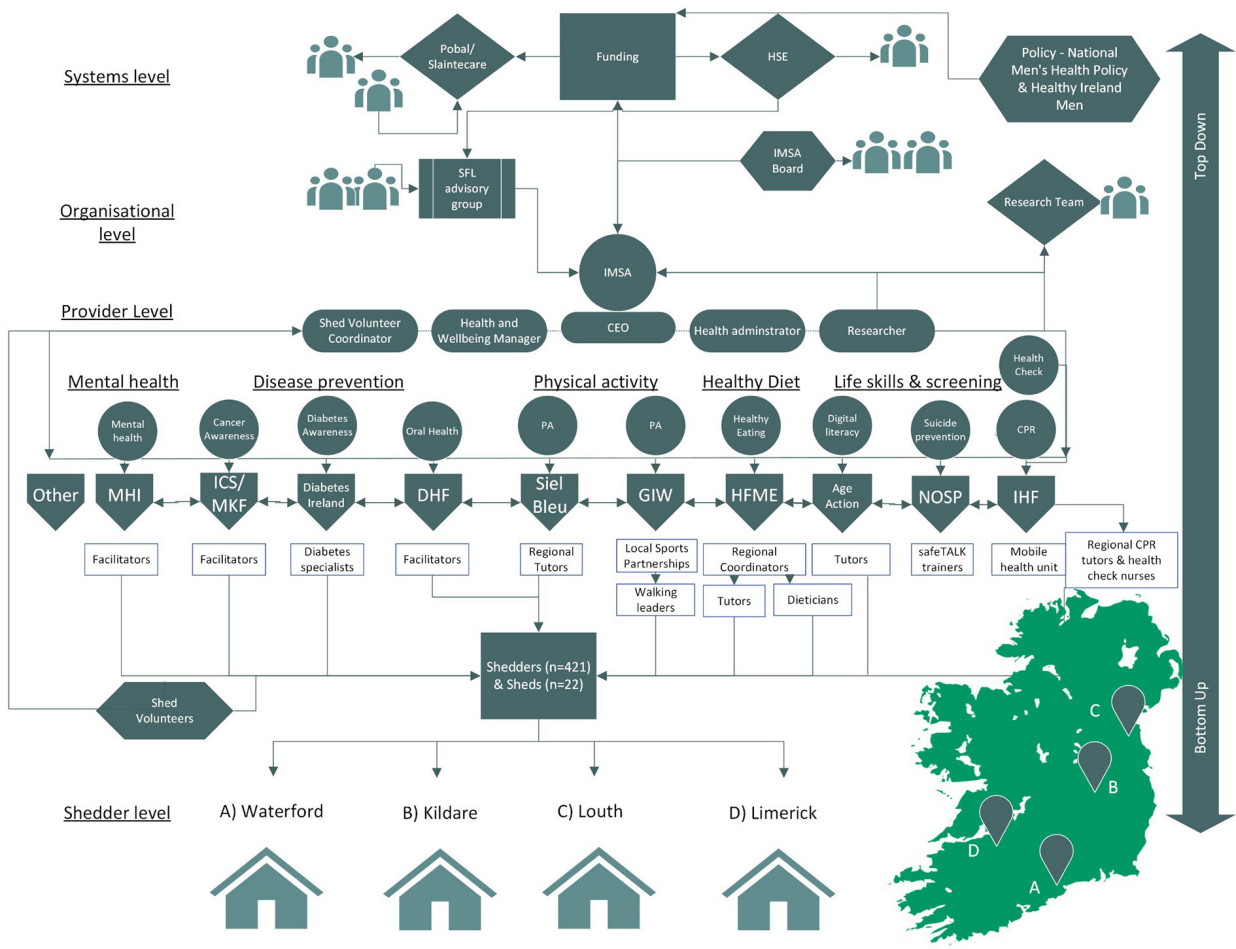
Sheds for Life (SFL) stakeholder map at systems, organization, provider and Shed level. IMSA, Irish Men's Sheds Association; SFL, Sheds for Life; HSE, Health Service Executive & Sláintecare (Funding SFL); Pobal (administration and management of Slaintecare SFL funding); IHF, Irish Heart Foundation (health check and CPR provider) ICS, Irish Cancer Society & MKF, Marie Keating Foundation (cancer awareness component); NOSP, National Office of Suicide Prevention (safeTALK component); DHF, Dental Health Foundation (oral health components) GIW, Get Ireland Walking & Siel Bleu (physical activity provider); HFME, Healthy Food Made Easy (HSE; healthy eating and cooking component); Age Action (digital literacy component); MHI, Mental Health Ireland (mental health component); Other (providers who may deliver new SFL content).

Data pertaining to SFL participation (attendance records, self-reported attendance, numbers who participated vs. numbers eligible) were triangulated to assess penetration. Cost-effectiveness was determined by comparing the costs (direct and indirect) of SFL to its benefits which were captured as the impact on quality-adjusted life-years (QALYs) derived from the short form-6D algorithm (35). Qualitative data were triangulated and analyzed using a framework-driven approach throughout implementation testing of SFL and refined using a constant comparison process applying the CFIR to identify barriers and facilitators. Focus groups and interviews were transcribed and, as per recommendations by the National Cancer Institute's White Paper on qualitative research in implementation science, a hybrid approach of thematic deductive and inductive analysis was used to identify barriers and facilitators and inform implementation strategies to address barriers and subsequent outcomes (37, 39). Initial codes were identified and data were then discussed with stakeholders throughout implementation of SFL in line with CBPR approaches, in order to ensure accuracy and identify strategies to address barriers to effective implementation. Figure 2 captures a stakeholder map of those involved in SFL delivery.

### Implementation testing and scalability assessment of Sheds for life

A detailed description of the implementation plan is outlined in the SFL protocol (19). In brief, the first implementation of the structured 10-week SFL implementation involved *n* = 22 Sheds and *n* = 421 Shedders across four counties in Ireland (two counties in March to May 2019 and two counties in September to December 2019) facilitated by *n* = 12 provider organizations and their subsequent regional deliverers (Figure 2 shows a conceptual map of SFL stakeholders). Participants (*n* = 421) were followed at baseline, 3, 6, and 12 months. These results are described in detail elsewhere and highlight both the efficacy of the SFL initiative in encouraging positive and sustained changes in health and wellbeing outcomes for Shedders (31), as well as supporting the case for scale-up (29). Baseline characteristics of participants also highlight that SFL was effective in engaging a cohort of HTR men (12). Implementation of SFL proceeded in the four counties outlined but due to the onset of COVID-19 Sheds remained closed for an extended period. Barriers and facilitators to further implementation within the changing implementation landscape were also monitored during this time. This process is described in detail below.

Insights into the determinants of implementation detailed below were then used to inform scalability assessment of SFL using the Intervention Scalability Assessment Tool (ISAT) (30). The ISAT is designed to assist policy makers, practitioners and researchers to determine the scalability of discrete health interventions. The ISAT is scored by a series of readiness questions to assist in identifying strengths and weaknesses across the domains. Domains in part A provide background information on the public health problem, the context within which it is proposed that the intervention will be scaled up, and a description of the intervention. Domains in part B consider implementation and feasibility factors relating to all aspects, including fidelity and adaptations, reach and acceptability, delivery settings and agents, as well as implementation infrastructure and training. Each question is scored from 0–3, where the minimum score for each domain is 0 and the maximum score is 3. In order to derive a final score for the domain, the average score across the questions is taken (if there is more than one question).

## Results

Results presented describe the process of implementation, the identification of implementation determinants (barriers and facilitators) as guided by the CFIR, identification of subsequent strategies to address barriers and how these steps informed implementation outcomes. Qualitative data will be used to support findings. The CFIR refers to barriers and facilitators as implementation determinants, as these determinants often have dual capacity to act as either a barrier or facilitator (32). Therefore, determinants in the context of this work mean contextual factors with potential to be either barrier or facilitator. The PRACTIS guide is used to structure presentation of results as per the four staged process of implementation (24); Step 1 summarizes the process of characterizing the implementation setting of SFL; Step 2 summarizes the process of identifying and engaging key stakeholders; Step 3; summarizes the process of identifying implementation barriers and facilitators which include a detailed summary of those identified and; Step 4; summarizes the process of addressing (where possible), barriers to implementation with a detailed description of implementation strategies used to address same. Figure 1 provides a flowchart of the evaluation process which is described in detail below.

### Step 1: Characterization of the Sheds for life implementation setting

Early familiarization with characteristics of the real-world implementation context aids planning and accountability that may enhance implementation efforts (24). Prior to the formal evaluation of SFL, members of the IMSA team consulted with Shedders at regional Shed “Cluster” meetings in 2017, which determined both an appetite for health and wellbeing in Sheds and signposted toward potential program content:

“*We started to take the input from what the men told us in terms of different areas, and the different areas that came up were the likes of the health checks, the physical activity, the walking, prostate cancer, mental health, various different topics like that which is what we currently have in SFL.*.”– Organization stakeholder

The IMSA then began to identify potential partners that they deemed suitable to deliver various aspects of health and wellbeing in Sheds, some of which had previously expressed interest in working with Sheds under their individual remits. This allowed *ad-hoc* piloting of what would later become components of SFL.

“*That gave us an insight as to what Shedders actually thought of having someone physically come out to the Shed consistently over a six week basis”* – Organization stakeholder

Previously described scoping work (10) highlighted that a key requirement for service provider organizations to work with Sheds was that they understood the ethos and Shed environment. This led to the development of a “Guidance for Effective Engagement with Men's Sheds” (GEEMS) manual and workshop, which were designed to promote understanding of the Shed environment and ethos for provider organizations and which remain a key implementation strategy of SFL. This was augmented by ENGAGE training—national men's health training for service providers seeking to work more effectively with men (40)—which was delivered to service provider organizations seeking to participate in SFL delivery.

“*There were a lot of organizations out there wanting to work with Sheds but they needed to understand what was the best way to engage with the men”* – Organization stakeholder

Following pilot testing of various SFL components, the IMSA expressed a desire to structure SFL into a suite of program offerings and the current research team then commenced the formal evaluation of SFL in collaboration with SFL stakeholders which began with characterizing the parameters of the implementation setting (24). This commenced with an iterative consultation process with the IMSA and research team exploring intervention design, adoption, delivery, sustainability and potential scalability as well as important multi-level contextual characteristics (24). Consideration was also given to evaluation design in terms of both effectiveness and implementation. This consultation process contributed to describing the Five P's for effective implementation as outlined by the PRACTIS guide (24). Table 1 outlines the output from characterization of the implementation setting.

**Table 1:** The Five Ps for effective implementation of SFL (24).

**The Five P's**	**Definition**	**Description**
People	The type and number of people that the intervention will reach, and the individuals that will be involved/required for implementation and scale-up	Considering capacity of the IMSA, research team and prospective provider organizations, consultation determined that a feasible approach would be to deliver SFL across four counties on a phased basis (two counties per phase) with the aim of engaging upwards of *n* = 350 Shedders *via* a clustered approach of circa *n* = 15 Sheds. The selection of counties was based on seeking a diverse representation of Shedders and Sheds in terms of size and geographical location (urban/rural). There was an overarching focus on engaging HTR men through a whole Shed approach. Shed support volunteers acted as a conduit on the ground to relay important information about SFL to Shedders during program delivery in conjunction with IMSA staff and the principal researcher. Sheds for Life was delivered by allied provider organizations whose ethos and goals aligned with the goals of the IMSA and who were deemed to be able to effectively respond to the needs of Shedders. This involved organizations who had participated in the GEEMS training and understood and respected the ethos and environment of the Sheds. This process was overseen by the IMSA in collaboration with academic partners.
Place	The setting/organizations that will be involved/required for implementation and scale-up	Sheds for Life consisted of a targeted intervention with the aim of delivery directly in the Shed settings. As Sheds are highly variable in terms of size and resources, alternative venues such as local community centers were sourced for those elements of SFL that could not be delivered in the Shed.
Process	The intervention or implementation process that will occur in practice	Sheds for Life sought to use gender-specific approaches to engage HTR Shedders with SFL. Recruitment involved an expression of interest process whereby Shedders retained a degree of autonomy and control by self-selecting into SFL. The principal researcher and health and wellbeing manager of the IMSA visited prospective participating Sheds to discuss the process of the SFL program and evaluation. Sheds for Life consisted of a 10-week, gender-specific intervention that commenced with a health check, weekly physical activity, healthy eating and mental health workshops, as well as optional components (e.g., suicide prevention, digital literacy, CPR, cancer, oral health and diabetes awareness) that allowed Sheds to tailor SFL to suit their individual needs.
Provisions	The resources that will be necessary to achieve intervention implementation and scale-up	•IMSA staff supported SFL recruitment and oversaw implementation (administration etc.). •Service provider organization staff delivered components of SFL in participating Sheds. •Recruitment materials were used to provide clarity (SFL expression of interest forms for Sheds). •Training workshop and GEEMS manuals were provided for providers of SFL. •SFL Handbook and component resources (leaflets, booklets, signposting etc.) were provided for participants. •Attendance records were given to providers to track attendance and attendance certificates were provided to participants. •Text-based reminder services were used and program calendars were supplied to participating Sheds. •Researcher gathered data one-to-one with Shedders and standardized protocols were used to measure outcomes at baseline, 3, 6, and 12 months. •Standardized protocols were also used to gather costs of implementation for economic evaluation. •Funding was provided by the Health Service Executive section 39 funding. Funding was also provided through individual grants and budgets of provider organizations with a view to securing alternative funding streams. The Irish Research Council's employment-based postgraduate scholarship funded the principal researcher's employment within the IMSA.
Principles	The underlying principles of the intervention (e.g., individual behavior change) and implementation process (e.g., building capacity for implementation) that will be used to scale-up in practice	**Intervention:** Capitalizing on the safe, familiar environment and social support within Sheds, gender-specific implementation strategies were used to engage “HTR” men with health and wellbeing. Using a co-design process, self-efficacy was enhanced through normalizing conversations about health and wellbeing in the Shed environment. Targeted outcomes included subjective wellbeing, diet, physical activity, mental health, social capital and help seeking. **Implementation:** Building on existing structures within Sheds, strengths-based approaches were used to maximize Shedders' involvement in the design and subsequent adaptations of SFL as it evolves. There was also an explicit focus on strengthening existing partnerships and identifying new partners who could potentially respond to evolving needs of Shedders. Identifying new funding opportunities to support SFL implementation was also a key target.

In summary, SFL was designed to build upon the inherent health promoting qualities of Sheds (delivery setting) while using participatory research methods to identify gender-specific strategies that would further enhance the reach of the program to HTR men (intervention population). The aim of the SFL design was to enhance health and wellbeing outcomes for Shedders while normalizing conversations about health for HTR men in Sheds through informal delivery and strength-based approaches (intervention characteristics). There was a strong emphasis in the recruitment phase on increasing the acceptability of SFL through trust and rapport building at Shedder level (intervention context). Evaluation methods were refined during this time to identify ways to monitor implementation for what was a complex multi-level intervention. This involved the previously outlined hybrid type 2 effectiveness-implementation design which also incorporated analysis of cost effectiveness. The implementation process also involved a partnership approach with all key stakeholders (Shedders, providers, IMSA, funders).

### Step 2: Identification and engagement of Sheds for life key stakeholders

The PRACTIS guide highlights the importance of participatory research to facilitate implementation and sustainability of complex community-based interventions (24). From the outset of the formal evaluation of SFL there was strong emphasis placed on identifying those aspects of the partnership between the multiple stakeholders that impacted most on SFL implementation and that would facilitate scale-up of the program. The IMSA also recognized the need for this stakeholder engagement as it was a critical success factor to ensure effective implementation of SFL:

“*Any partnership has three main columns…it starts with the men primarily, then its IMSA, then it's the partner organization and the three have to work in tandem otherwise it doesn't flow”-* Organization stakeholder

The structured format of SFL was designed to engage key stakeholders from the outset. At a top-down systems and national men's health policy level (41, 42), the need for community-based men's health programs such as SFL was clearly mandated. These priorities also aligned with the National Health Service Executive's (HSE) priority programs. Thus, core components of SFL aligned with the key pillars of the Healthy Ireland Framework including healthy eating, physical activity and mental health (42). This was a key facilitator of stakeholder engagement at systems (HSE) level and helped leverage funding to support core staff at the IMSA to oversee delivery of SFL:

“*Over the last couple of years we have funded the health and wellbeing initiatives in Sheds and Sheds for Life is a realization of that, the realization of an actual program of work. Not just giving information but engaging with men” –* HSE stakeholder

The SFL advisory group was consulted quarterly and brought considerable experience in men's health policy, practice and community development work to help guide and shape the evaluation and implementation of SFL. This further guided the actions of what would be structured as the SFL stakeholder group.

At the organizational level the first author was positioned within the IMSA for the duration of the research and worked closely with the health and wellbeing manager to promote effective implementation and co-production of SFL in line with evidence on men's health practice, while also ensuring that the implementation strategy aligned with existing practices and infrastructure.

Acknowledging how critical provider organizations (POs) were to the delivery of SFL, the IMSA spent time building relationships with multiple POs prior to the formal evaluation (see Figure 2). The implementation process focused on strengthening these partnerships through the formation of a structured stakeholder group. Provider organizations were consulted throughout the implementation process about implementation strategies, assessment of the implementation environment and they participated in the evaluation process to promote pragmatic and context-driven research. New providers were invited to join the SFL team in response to identified Shedder needs prior to implementation of SFL. In the absence of large-scale funding for SFL, priority was placed on identifying partners that understood the need for SFL. These providers were sought with a view to adopting a sustainable delivery model under real-world conditions where providers could undertake delivery as part of their routine work plans - as opposed to seeking short-term (and often unsustainable) grant funding to get SFL established. This meant that a prudent approach was needed in matching Sheds' needs with SFL offerings. The participatory approach with providers was therefore critical to sustained engagement:

“*I suppose one of the strengths of SFL is the fact that the partner organizations invested their time and their resources in SFL without actually getting any financial return on it”* – Organization Stakeholder

While there were no financial incentives, stakeholders had an active role in the development of evaluation tools (questionnaires) to encourage adoption where evaluation of each POs component of SFL was a key engagement strategy:

“*The evaluation, I think it's an important one and I think that's going to be important for us, I think from a research perspective as well to be involved in that”–* SFL Provider

Moreover, the priorities of POs aligned with those of the IMSA and SFL in reaching HTR men which is a noted challenge in community-based work, and thus SFL provided opportunities to connect and foster long-term buy-in and support.

“*I got involved with the Men's Sheds because over 80% of our participants are women. So, we weren't reaching men, we weren't reaching that cohort, so we identified a male group within the Sheds Association to do that.” –* SFL provider

Shedders were viewed as key stakeholders throughout the evaluation process of SFL as both hosts of SFL in the Sheds setting and intervention users. While SFL had a top-down policy directive, it mostly evolved as a bottom-up initiative to address a particular need within Sheds. Considerable time was spent in the Sheds as outlined in the methods to capture Shedders' experiences of SFL in practice as well as to co-design the structure and delivery of SFL. Sheds for Life was promoted as a program “*For Shedders by Shedders”* with Shedders having a crucial role in the identification of barriers and facilitators at Shed level. This engagement and co-design process were critical to acceptability and appropriateness of SFL implementation (these strategies will be further described in subsequent sections (see Table 3):

“*There was a genuine openness from you to hear ‘well what was your experience?' and ‘how did it go?”' –* SFL participant

Table 1 provides details on the structure of SFL with a further detailed breakdown available in the SFL protocol (19). Findings from scoping work (10) in consultation with key stakeholders guided the decision to structure SFL as 10-week program. This format was viewed acceptable by POs and the IMSA as it was long enough in duration in terms of the practicalities of delivery and encouraging positive and sustained behavior change. Crucially, from Shedders' perspective, it also respected the fluid nature of Sheds in which a longer program might conflict with Shed routine. Moreover, this structure was pragmatic enough to consider whether SFL was feasible in the real-world, capricious Shed environment while prioritizing future sustainability within existing funding structures. This structure and format were also informed by what worked in other programs in Ireland with similar cohorts of men within community settings (43). In terms of its design, the flexibility of SFL such as the optional components provided Shedders with an opportunity to tailor SFL to suit their needs while also instilling a sense of autonomy and control:

“*I liked the fact that it was modular and that you consulted people about their particular interest beforehand*“ – SFL participant

In summary, SFL emerged from an invested process of engagement, consultation, relationship building and pilot testing. These efforts seeded partnership networks that understood the processes and recognized the value in engaging men with health. This was an important consideration at a time when Sheds had been earmarked as settings that facilitated access to HTR men and where expectations placed on Sheds to expand into formal healthcare delivery may have caused tensions within Sheds (10). While it was recognized that the implementation evaluation would lead to refinement of SFL, meaning its structure could ultimately evolve, it was understood that this process of delivery and vested partnerships were the crux of its sustainability:

“*The partners add a different dimension to it [SFL] because we can't be experts in all aspects of men's health. We were able to use their expertise, use our own expertise and understanding of what works with Men's Sheds to package SFL in such a way that it got the men's interest and kept them engaged across the program as well*.” - Organization stakeholder.

### Step 3: Identification of implementation determinants (barriers and facilitators)

**Figure 3:**
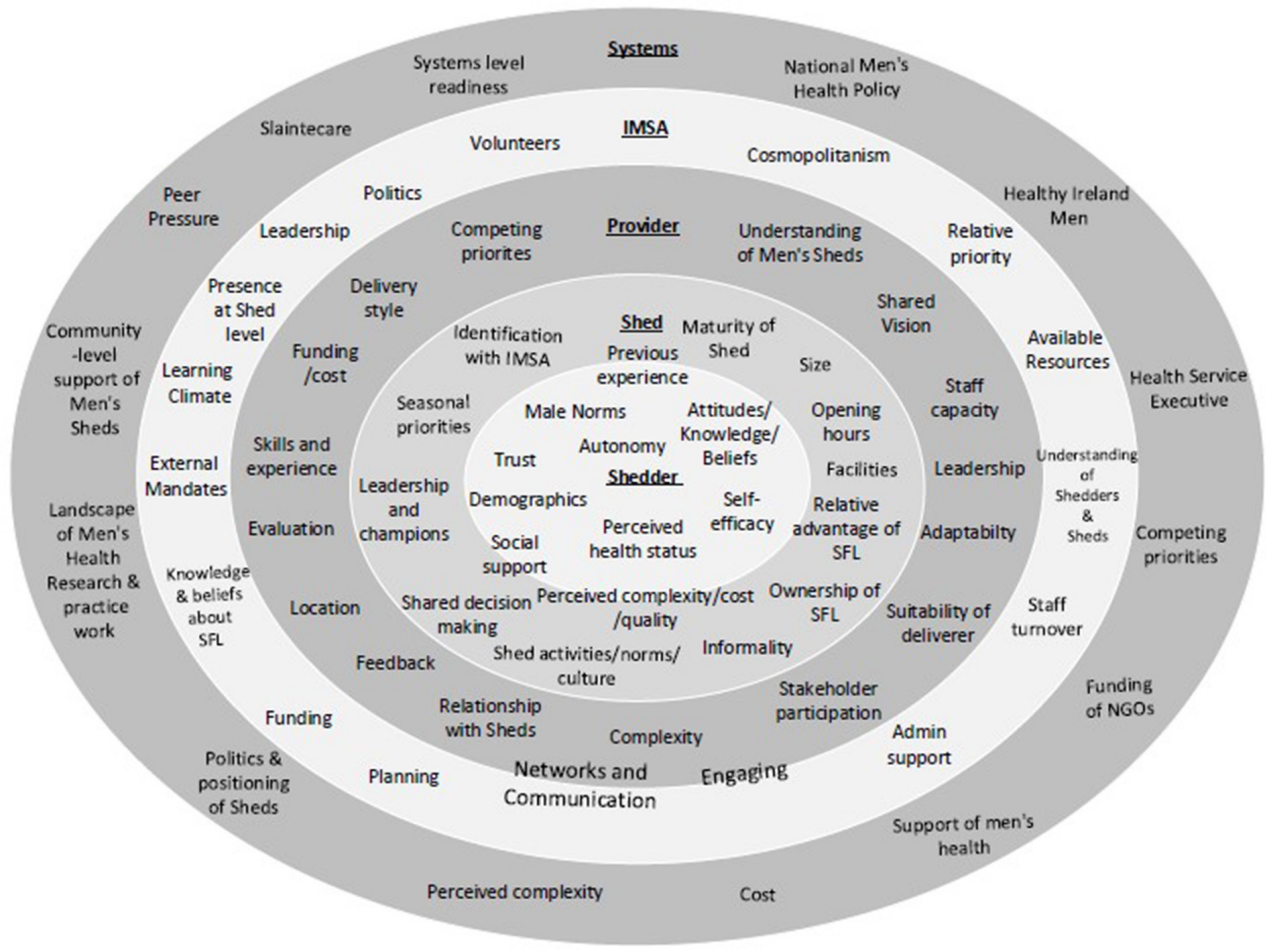
An adapted ecological model of SFL implementation (24).

The purpose of identifying contextual barriers and facilitators to SFL implementation was to enhance implementation effectiveness through integration of research findings into practice (24). Barriers and facilitators were identified throughout SFL implementation *via* the multiple data collection techniques outlined at Shedder, Shed, PO, organization and systems level. The CFIR was used as a guide to group determinants at each level of implementation- some of which influenced all ecological levels. Table 2 describes the determinants to SFL implementation as guided by the CFIR with adaptations that were also context-specific. Figure 3 also conceptualizes the most prominent determinants in an ecological model of SFL implementation.

**Table 2:** Determinants of SFL implementation across individual, provider, organizational and systems level as per CFIR (32).

**Identified determinants**	**Description**
**Shedder (user) & shed (inner setting) level**	
Personal attributes (Male norms)	Shedders' perceptions of how acceptable it is for men to discuss or engage with health issues
Perceived health status	Shedders who overestimate their health status may underestimate their perceived need of SFL (12)
Demographic of Shedders	The likelihood of HTR men engaging with SFL/the diversity of backgrounds as a facilitator to engaging HTR Shedders
Knowledge and beliefs about the intervention	Shedders' attitudes toward, and value placed on SFL, as well as familiarity with facts, truths and principles related to SFL
Previous experience	Shedders past experiences of “health” programs as an influencing factor to engagement with SFL/Sheds past experiences of external providers delivering health components in Sheds
Autonomy	The importance Shedders place on maintaining a sense of autonomy and control/ Implementing SFL while respecting Shedder autonomy—no compulsion to undertake an activity
Trust	The need for Shedders to become familiar with and trust POs prior to engagement
Self-efficacy	Shedders' belief in their own capability to participate in SFL
Perceived complexity/cost/quality	Shedders' perception of the difficulty and intricacy of participating in SFL as well as perceived cost Sheds' perception of the cost of the time commitment and potential disruptiveness of SFL to Shed routine Sheds' perception of how well SFL is presented and subsequent belief it will lead to desired outcomes
Relative advantage of SFL	Shedders' perception of the advantage of participating in SFL vs. no intervention
Social support	Shedders' sense of motivation and safety participating with fellow Shedders Level of social support in Sheds to encourage sustained engagement (peer mentoring and peer support)
Shared decision making	Whether a Shed decides to participate in SFL or not based on group consensus or select individual(s)
Leadership (opinion leaders) and champions	Shedders who have formal or informal influence on the attitudes and beliefs of other Shedders with respect to SFL Perceptions that leaders in Sheds who act as point of contact have about SFL and their choice to filter messages about SFL to Shedders Shedders who dedicate themselves to overcoming resistance or indifference that SFL may provoke in Sheds
Identification with the organization	How Shedders perceive the organization (trust vs. mistrust) and their relationship and degree of commitment to the IMSA
Intervention source—Ownership of SFL	Shedders' identification of SFL as being developed by Shedders or externally developed
Compatibility—Shed activities, norms and culture	The compatibility of norms, culture and nature of activities and work systems in different Sheds with SFL (e.g., workshop vs. social focus)
Compatibility—Seasonal priorities	Shed activities increase during certain periods of the year (e.g., Christmas, summer) which may impact acceptability of SFL
Compatibility—Informality of Sheds	The degree of tangible fit between SFL and the informal nature of Sheds where informality is important to ethos while attendance and participation is sporadic
Structural characteristics—maturity, size, opening hours, and facilities	The maturity of Sheds as an influencing factor in recognizing the value of SFL (e.g., older vs. newer Sheds) The physical size of the Shed and number of Shed members to accommodate SFL The sporadic opening hours of Sheds and ability to schedule SFL activities The facilities of Sheds to accommodate SFL (e.g., running water, kitchen facilities)
**Provider level**	
Shared vision	Provider organizations who have a desire or mandated remit to engage men in their health endeavors
Relative advantage—evaluation	The POs perception of the advantage of their component of SFL being externally evaluated
Compatibility—competing priorities	The degree of fit of SFL within the PO among other priorities
Complexity	POs perception of the difficulty of implementation and the intricacy and number of steps to implement (e.g., identifying deliverers, coordination across locations)
Shedder needs and resources—understanding of Men's Sheds	The extent to which Shedders' needs as well as barriers and facilitators to meet those needs are accurately known and prioritized The extent to which the ethos and environment of Sheds is accurately known and respected during delivery
Shedder needs and resources—delivery style	How providers deliver their component of SFL (informal vs. formal; facilitative vs. didactic style)
Shedder needs and resources—relationship with Sheds	Amount of time invested by providers to build relationships with Shedders
Patient needs and resources—suitability of deliverer	How the program deliverer is perceived by Shedders in terms of age, gender, experience
Self-efficacy—skills and experience	Deliverers' beliefs in their own capabilities to execute courses of action to deliver SFL component effectively
Available resources—staff capacity	The capacity and number of staff available to deliver SFL components
Available resource—location	The capacity to deliver SFL components in multiple locations and regions
Available resources—funding/cost	The level of funding available for POs to dedicate to on-going delivery The perceived cost and return on investment of SFL for organizations
Opinion Leaders—Leadership	Individuals within the PO who have a formal or informal influence on the attitudes and beliefs of their colleagues with respect to implementing SFL
Networks and communication	The nature and quality of formal and informal communication with the PO, organization, deliverers of SFL
Engaging	The POs capacity to involve appropriate individuals in the implementation of SFL through education and training
Adaptability	The degree to which the PO can adapt, tailor, refine of reinvent the SFL component to suit Shedder & Shed needs
Access to knowledge & information—feedback	Iterative feedback from the evaluation of SFL for POs to incorporate into work tasks
Cosmopolitanism—Stakeholder participation	Opportunity for POs to have ownership of SFL while networked with other external organizations
**Organizational level**	
External mandates & funding	Men's health policy which recommends delivery of tailored men's health programs Pressure by external mandates to implement SFL within specific timeframe Amount of systems level funding received to implement SFL
Available resources (admin support, money, time, staff)	The capacity of the organization to dedicate resources for ongoing SFL implementation The capacity and number of staff within the organization to implement SFL effectively The capacity of the organization to dedicate required admin support to coordinate SFL Staff turnover within the organization
Understanding of Shedders and Sheds	The extent to which Shedder needs as well as facilitators and barriers to meet those needs are accurately known and prioritized by the organization (e.g., new staff)
Knowledge and beliefs about SFL	Attitudes toward and value placed on SFL as well as familiarity with facts and principles related to SFL—particularly new staff
Relative priority	Perception of the importance of implementation of SFL within the organization among competing priorities
Learning climate	Transparent communication where team members feel they are essential, valued and knowledgeable partners
Organizational incentives and rewards	The capacity of the organization to retain staff and key implementers of SFL through incentives (promotions, salary)
Leadership	Key implementers in the organization who understand the principles of SFL, recognize its value and positioning within the wider system and can advocate for needs at Shedder level The role of the Shed support volunteers in encouraging engagement with SFL and acting a conduit between the organization and Sheds
Networks and communication (Politics & presence)	The nature and quality of social networks and quality of formal and informal communication with the organization and between the organization and Sheds Capacity of the organization to have a ground-level presence with Sheds to foster positive perceptions of the organization
Cosmopolitanism	The degree to which the organization is networked with and maintains relationships with other stakeholder organizations
Engaging	Capacity of the organization to attract and involve appropriate stakeholders in the implementation of SFL through combined strategies (social marketing, training) and maintain momentum for implementation Capacity of the organization to involve Shedders in the use of SFL through combined strategies (education, gender-specific approaches, role modeling)
**Systems level**	
Systems level readiness	The impact of the COVID-19 on readiness to implement SFL due to the complexity of disruption at all levels of the implementation environment
Evidence strength and quality	Rich landscape of men's health research and practice work supporting the belief the SFL will have desired outcomes Dedicated men's health training (ENGAGE) to build PO capacity
National men's health policy	Ireland's national men's health policy championing men's health practice—encouraging buy-in
Healthy Ireland Men	Strategic framework for men's health under the implementation of Healthy Ireland—national framework for population health
Support of men's health	Systems level understanding of the need for gender-specific men's health approaches and the positioning of men and masculinities
Competing priorities	Ability to secure support for SFL amongst competing priority areas
Community-level support of Men's Sheds	Attitudes toward, recognition and value placed on Sheds in communities
Politics and positioning of Sheds	The tangible fit of Sheds within the remit of different government departments and how they align with government priorities
Perceived complexity	Perceived difficulty by decision makers of the difficulty of implementing SFL considering the intricacy of multiple stakeholders and variables
Cost	The perceived cost of implementing SFL compared to other interventions as a funding determinant
Health Service Executive	The capacity of the HSE structures to support sustainability of SFL
Slaintecare	Ten-year program to transform health and social care services—shift of services to community setting & capacity to support SFL
Funding of NGOs	Level of adequate and stable funding available for NGOs providing important public services
Peer pressure	Pressure for other organizations at local or regional level; to implement health and wellbeing in Sheds in silo which may detract from SFL

#### Determinants at Shedder and Shed level

Table 2 provides a description of the implementation determinants at Shed and Shedder level. Table 3 outlines implementation outcomes, their influencing determinants as well as strategies to address barriers toward implementation at Shed and Shedder level. Alongside the scoping work (10) which highlighted the importance of respecting the Shed environment as a core determinant to Shedders' acceptability of SFL, SFL was built upon an evidence base of men's health research and practice work that employed gender-specific strategies to engage men with health while utilizing the Shed as a foundation for SFL (40, 41, 43–45). This helped to engage HTR Shedders in a familiar and safe way and to overcome barriers at the individual level such as previous adverse experiences with engaging in health and Shedders perceptions of socially acceptable ways that men should behave in relation to discussing and engaging with health issues:

“*Women talk about their health, they talk about their feelings whereas fellas, you're a man! You don't talk about it.”* – SFL participant

**Table 3:** What strategies enhance implementation in SFL? Outcomes of SFL, influencing determinants and strategies to address implementation barriers.

**Implementation** **outcome definition (33)**	**Measurement**	**Level (33)**	**Influencing determinant(s)**	**Strategies to address barriers to implementation and enhance outcomes**
Acceptability *Acceptability* is the perception among implementation stakeholders that a given treatment, service, practice, or innovation is agreeable, palatable, or satisfactory.	Stakeholder consultations & Interviews	Provider	Shared vision Relative advantage Compatibility & Complexity	•*Allied partnership approach:* SFL was delivered and designed in collaboration with POs who clearly perceived the advantage of implementing SFL through a shared vision, aligning with their organization in accessing a HTR group of men. SFL responded to the increasing calls *by national policies* to implement gender-specific strategies that engage HTR men with health which were applicable to PO's. •*Stakeholder engagement:* POs were continually engaged to promote shared decision making in the implementation of SFL to limit perceived complexity.
	Focus groups, Interviews & Ethnography	Shedder	Personal attributes Knowledge & beliefs about SFL Previous experience Trust Perceived complexity Relative advantage Identification with organization Ownership Compatibility & structural characteristics	•The intervention was designed and refined with *underlying gender-specific approaches* that enhanced the organic health promotion in Sheds. •*Targeted intervention:* delivered in a targeted way by bringing SFL to the Sheds and delivering the majority of its components directly in the Sheds natural environment or other local community setting, which were viewed as *familiar, safe and non-clinical, environments* for Shedders. This removed barriers toward participation and made participation *convenient*. •*Expression of interest and Active Recruitment:* Sheds were encouraged through *shared decision making* to opt into SFL participation—it was not foisted on Shedders. When Sheds expressed interest the researcher and health and wellbeing team in the IMSA visited each individual Shed and discussed the process of SFL in an *informal* way, reducing perceived complexity, *building trust and actively recruiting* individual Shedders and addressing their concerns. This strategy also aimed to enhance the relationship and *sense of trust between the IMSA and Sheds*. •*Co design process:* SFL was described to prospective participants as a program “for Shedders by Shedders”. Prospective participants were encouraged to see themselves as *pioneers*, actively shaping the program through their participation and paving the way for future delivery and scale-up. Reinforcing Shedder' *sense of ownership* was designed to build safety and trust, and to reassure participants that SFL was not being implemented to undermine the routine environment and ethos of the Sheds. Involving Shedders in the implementation process also facilitated *access to local knowledge and resources* for SFL implementation while building relationships enhanced the sense of *social capital* that positively influenced implementation.
Adoption *Adoption* is defined as the intention, initial decision, or action to try or employ an innovation or evidence-based practice.	Stakeholder consultations, interviews & observation	Provider	Shared vision Understanding of men's health Opinion leaders Stakeholder participation	•POs who *understood the value* of implementing SFL in Sheds and understood the need for gender-specific approaches were engaged in the *stakeholder process*. •*Opinion leaders* within the POs were valuable in *building momentum* to join the partnership network. •The *Participatory Research Approach* where all key stakeholders acted as decision makers in SFL design and implementation that is *built upon evidence-based practice* was a key facilitator in adoption at PO level.
	Consultation & Observation	Organization	External mandates & funding Understanding of Shedders Relative priority Leadership	•The implementing organization responded to both *top down* (policy and funding incentives) and *bottom up* (Shedder needs) calls to deliver health promotion in Sheds. •Sheds for Life was viewed a priority program in the organization. •*Leadership* from key implementers (health and wellbeing manager and researcher) who *worked in partnership* to strengthen implementation enhanced the perceived *importance of SFL among other competing priorities*.
	Administrative data, Focus groups, Interviews & Ethnography	Shed setting	Trust Social support Self-efficacy Leadership Shared decision making Autonomy Knowledge & beliefs about SFL	•*Trust and relationship building* through time spent in the Shed setting at recruitment phases was a key enabler of adoption within the Sheds. The *co-design* process facilitated reassurance among Shedders that SFL would remain *respectful of the Shed* environment and the *autonomy* of Shedders. •Shed support volunteers or *champions* played a key role in encouraging Sheds to try SFL. *Designated contact points* in each Shed act as a conduit between Shedders and program delivery. •*Leaders within Sheds* were also pivotal to adoption and engagement at Shed level and time was spent with identified leaders during Shed visits and national Shed volunteer coordinator events to *ensure that key influencers understood the value of SFL* for Sheds. *In person visits by the recruitment team* to Sheds were also a critical facilitator to adoption as it ensured that *messages about SFL were disseminated to all Shedders* (rather than one influencer who may not intend to adopt) and this encouraged shared decision making among Sheds. •SFL *capitalized on the organic health promotion* that occurs through the already existing *social support between Shed members* in Sheds. More reticent Shedders were encouraged to participate by *Shedders with a higher sense of self-efficacy*. •*Use of “Hooks”:* A free comprehensive health check at the beginning of SFL is a critical incentive to engage men in the SFL program alongside other life-skill components such as CPR.
Appropriateness Appropriateness is the perceived fit, relevance, or compatibility of the innovation or evidence based practice for a given practice setting, provider, or consumer; and/or perceived fit of the innovation to address a particular issue or problem.	Focus groups, Interviews & Ethnography, participatory research	Shedder & Shed setting	Compatibility Ownership Autonomy Perceived complexity Structural characteristics	•*Male specific:* An underlying principle of SFL was to deliver in the male-only environment of the Shed in the company of like-minded men which promotes a sense of safety and motivation through *friendly competition*. •SFL was co-designed as a *tailored intervention* with core components but allows autonomous decision making over adaptable or supplementary elements which the Sheds can “self-select” into. It is continually refined in collaboration with Shedders to respond to their needs. •*Respecting the Shed environment:* The co-design process and early testing of SFL determined characteristics of Sheds to be key determinants of implementation (see Table 2). •*Timing:* Shedders are also recommended to *designate a specific day of the week* to dedicate to SFL so that it does not encroach on the typical routine of the Shed. A *readiness assessment* also informs whether SFL is suitable for a Shed at that time in terms of competing priorities, resources or maturity (e.g., newer Sheds may see SFL as an opportunity to build relationships whereas Sheds heavily established in workshop based activities may view SFL as detracting from primary Shed aims). During assessment by implementers at recruitment phase, Shedders with few resources or members may *use nearby community resources or join with another Shed* to participate in SFL. As determined *via* co-design, SFL also aims to be *implemented during times that are conducive with the Shed environment* such as spring or autumn avoiding busier project periods for the Sheds such as Christmas or summer.
				•Sheds for life was *delivered free of charge* to eliminate cost barriers for Shedders. •*Autonomous Participation:* Alongside the expression of interest process, individual Shedders are asked to participate in as much of SFL as possible while recognizing and respecting that other life commitments happen. The central goal of SFL is to enrich, not undermine the Sheds already health enhancing environment and so alongside ongoing collaboration with Shedders, participants of SFL are also *guided not to overburden* themselves by committing to too many SFL components. •*Structure, Clarity & Supportive Resources:* As perceived complexity was a noted determinant, participants receive *supportive resources* during SFL such as dedicated SFL and Healthy Food Made Easy handbooks as well as material on mental health and other various components. Participants are visited by the recruitment team to explain the process of SFL and also receive *text reminders and prompts* during SFL delivery along with *program calendars and screening appointment cards*.
	Stakeholder consultations, interviews & observation	Provider	Complexity Delivery style Relationship with Sheds Networks & communication Adaptability	•Both *formal and informal meetings* with stakeholders were used to limit complexity for POs and the IMSA coordinated and oversaw delivery of individual SFL components. •*Credibility and capacity building:* POs were seen as part of an allied partnership network bringing expertise from a variety of credible and informed sources thus enhancing perceived quality of SFL in Sheds. POs also participated in GEEMS and ENGAGE training for effectively working with men. •*Adaptability:* POs through stakeholder engagement were encouraged to tailor their components to suit both the cohort of men and the Shed environment. •*Informality of Sheds:* SFL was refined to be delivered in an *informal, interactive and relaxed way with a conversational tone*. Through iterative feedback POs of SFL were encouraged to spend time *building rapport and trust with participants prior to delivery of SFL components*. Informal delivery respects the ethos of the Sheds and facilitates comfort and active participation. •*Strengths-Based Approach:* SFL aims to be delivered using a strengths based approach where facilitators *utilize the capacity, skills and knowledge of the men* while demonstrating empathy and respect and *using positive, non-stigmatizing or non-judgemental language and tone*.
		Organization	External mandates & funding Understanding of Shedders Engaging	•*Men's health policy* was an enabler to leverage support for SFL. Involving Shedders in the decision making process meant the organization was best positioned to understand and prioritize Sheds and Shedder needs. •The *sustained engagement* of appropriate stakeholders maintained momentum for implementation.
Implementation Cost *Cost (incremental or implementation cost)* is defined as the cost impact of an implementation effort.		Provider	Available resources Complexity Adaptability Relative advantage	•While delivering SFL incurred additional time and monetary cost in terms of adaptations and delivery—*POs that were able to incorporate SFL into part of their routine delivery* could facilitate implementation with the advantage of accessing a group of HTR men for their own organization.
		Organization	Funding Available resources Cosmopolitanism Engaging	•*Sustainable funding* would be a key determinant of SFL implementation and maintenance of partnerships. The capacity of the organization to *network and engage key stakeholders who could support SFL delivery* was a key enabler of supporting implementation costs. The *evaluation* of SFL was a key facilitator in highlighting the impact and cost-effectiveness (35) of SFL which gave the organization *leverage to engage funders* for substantial funding for SFL (e.g., Slaintecare).
Feasibility *Feasibility* is defined as the extent to which a new treatment, or an innovation, can be successfully used or carried out within a given agency or setting	Stakeholder consultations, interviews & observation	Provider	Compatibility Adaptability Shedders needs & resources Available resources Complexity Leadership Engaging	•The *participatory research approach, pilot testing and partnership building* were key facilitators in ensuring feasibility at provider level. •Feasibility has been demonstrated through *measurement of impact on health and wellbeing outcomes* of participants up to 12 months (31).
	Stakeholder consultations, interviews & observation	Organization	Available resources Understanding of Shedders Relative priority Leadership Cosmopolitanism Engaging	•The *partnership approach to SFL alongside the leadership at organizational level and the refined research approaches* were key facilitators to feasibility of SFL at organizational level.
	Focus groups, Interviews & Ethnography	Shed setting	Compatibility Structural characteristics Intervention source Leadership	•The co-design process where *SFL was viewed as “internally” developed* was critical to ensure that SFL was compatible and appropriate for Sheds. The initiative was also based upon evidence-based practice that engages men at community level, previous piloting of SFL informed the current strategy. •Leadership was also a key facilitator at Shed level to ensure successful implementation of SFL. •The implementation team endeavored to deliver SFL directly in the Shed setting, *where resources were lacking in Sheds*, kits including portable ovens and kitchen *supplies were sourced to facilitate delivery* of HFME within the Shed.
Fidelity *Fidelity* is defined as the degree to which an intervention was implemented as it was prescribed in the original protocol or as it was intended by the program developers	Stakeholder consultations, interviews & observation	Provider	Self-efficacy Knowledge and beliefs about the intervention Available Resources Adaptability Access to information & knowledge	•Fidelity was viewed as an important outcome for SFL as it moved across Shed settings. *Fidelity was facilitated by consistent use of POs*. *Stakeholder engagement* was used to ensure deliverers at ground level understood the underlying principle of SFL and GEEMS and ENGAGE training was made available. •*Iterative feedback* though the participatory research approach was used to address any identified issues with fidelity. It was recognized through the process evaluation that *adaptations at local level were necessary for fidelity* of SFL and there were facilitated through a consultation process.
Penetration Penetration is defined as the integration of a practice within a service setting and its subsystems.	Administrative data & observation	Shed setting	Knowledge & beliefs about the intervention Perceived complexity Leadership Ownership Compatibility	•*Penetration of SFL at Shed level was encouraged through multiple implementation and gender-specific strategies* outlined. Penetration in phase 1 delivery was captured by assessing the number of Shedders in the participating Sheds who eligible to attend vs. the number of Shedders who enrolled in SFL. •*Assessment of the baseline profiles of Shedders also assessed whether SFL was reaching the HTR cohort within Sheds* (46)
	Consultation and observation	Organization	External mandates & funding Knowledge and beliefs about the intervention Relative priority Leadership	•*Penetration at the organizational level was facilitated by the evaluation of SFL* which demonstrated the efficacy and cost-effectiveness of the approach. Sheds for Life was recognized by the organization as a priority program which is capable of leveraging support for Sheds at a systems level. Leadership of key implementers was an important enabler to champion SFL at organizational level.
Sustainability *Sustainability* is defined as the extent to which a newly implemented treatment is maintained or institutionalized within a service setting's ongoing, stable operations	Consultation and observation	Organization	External mandates & funding Available resources Relative priority Organizational incentives and rewards Leadership Networks and communication Cosmopolitanism Engaging	•*Sustainability of SFL is facilitated by leadership at organizational level* and the necessary resources needed to maintain momentum among stakeholders across implementation levels. The ability of the organization to retain key implementers as well as the support and funding at systems level are key determinants of sustainability.

The recruitment phase of SFL was a critical facilitator to implementation as this period allowed trust and relationship building which was key to acceptability and adoption of SFL by Shedders:

“*We had sort of a trust and faith in the program because it wasn't just a fob” –* SFL participant

The time spent in the Sheds by the researcher and health and wellbeing team was also critical at this point in terms of identifying the local contextual factors and structural characteristics within Sheds that needed to be considered in molding SFL to suit individual Sheds. This also facilitated an understanding of the intricacies of the different operational systems of individual Sheds which determined that SFL should be seasonal (autumn & spring) and that SFL would not be appropriate to Sheds currently engaged in demanding project work. This was an important finding in terms of respecting the environments of Sheds and generating positive perceptions of SFL among Shedders rather than it being seen as an innovation foisted upon them. Moreover, this was a critical time to identify formal and informal opinion leaders in Sheds that would facilitate buy-in, to ensure whole Sheds received adequate communication about SFL and to dispel misconceptions about SFL. The relationships within Sheds were also key determinants to implementation of SFL. In particular the social support and informal peer mentoring among Shedders was key to supporting and engaging more HTR Shedders. Moreover, Shedders recognized the value of SFL in enriching the social support within Sheds by bringing Shedders together:

“*It became more of a social aspect than we had had and I think bonds might have been strengthened a bit because of the course and I think it was good for the Shed”* – SFL participant

The co-design process, targeted (delivered directly in Sheds) delivery and modular format of SFL instilled a sense ownership, autonomy and control over SFL within Sheds which was key to acceptability and adoption. Shedders recognized the value of SFL being implemented directly in Sheds which was key for engagement of HTR Shedders:

“*Sheds for life worked because it came to us. We wouldn't be as forthcoming as to go to it. That's men for you.”* – SFL participant

Overall at Shed and Shedder level, the implementation of SFL demonstrated feasibility and impact in terms of positive and sustained health and wellbeing outcomes among participants as outlined in a SFL outcomes paper (31). Moreover, SFL successfully transferred across Shed settings demonstrating its transferability and feasibility for scale-up in this regard. In terms of penetration the design of SFL demonstrated that it was capable of reaching the target cohort of HTR men within Sheds. Penetration has been highlighted elsewhere (46) but was assessed *via* administrative data and attendance records. This determined that of the *n* = 565 Shedders eligible to participate in SFL, *n* = 421 enrolled, a reach rate of 75%. The adoption of SFL at Shedder level was facilitated by the gender-specific strategies and co-design process where Shedders worked in partnership with the researcher and IMSA team to identify best practice at Shed level:

“*I think that what it is here [SFL] is whatever we are going to do we are going to do it together and I think it's the sense of togetherness” –* SFL participant

The informal delivery approach was a key facilitator to sustained engagement of Shedders. Overall the approach was appropriate to the Shed environments, which are highly variable informal settings, and implementation requires careful consideration of the multiple determinants outlined. It is also important to note that these variables do not remain fixed and evolve with Shedder needs. Therefore, in order for further implementation of SFL to remain impactful and appropriate to the Shed setting, the determinants and strategies outlined are critical to its sustained success most notably investment in relationships and partnerships with Shedders.

#### Determinants at provider level

Partnerships are key to the successful implementation of SFL in terms of both delivery of SFL content but also in terms of championing the wider SFL movement and providing valuable insights to address facilitators of, and barriers to SFL within the stakeholder engagement process. Fostering partnerships with those who shared the vision and recognized the relative advantage in accessing a group of HTR men in their health promotion endeavors was key to acceptability and initial adoption of SFL at PO level. Moreover, the administrative assistance by the IMSA in terms of coordination and delivery of SFL limited complexity for POs thus enhancing acceptability. The stakeholder engagement instilled a sense of ownership among POs of SFL and, alongside the enjoyment and sense of reward offered from working in Sheds, adoption of SFL remained high for POs throughout implementation of SFL, which is demonstrated by their continued and sustained engagement:

“*You're going into a formed group. They've already gelled and are ready, and primed for information and once it's facilitated well - it's just a pleasure to deal with that group you know, knowing that they're at risk and the messages that we want to give.”-* SFL deliverer

The stakeholder engagement, real-time feedback and discussion facilitated by the research team and the IMSA was a key strategy to overcoming barriers in relation to fidelity and adaptations needed to strengthen delivery such as ensuring an informal delivery style, suitable deliverers for Sheds and encouraging relationship building among POs and Shedders. Indeed, the informal nature of Sheds can present challenges to implementation (e.g., sporadic attendance) and was a key discussion point throughout stakeholder meetings. However, SFL was refined to be delivered in an informal, interactive and relaxed way with a conversational tone. Through iterative feedback POs of SFL were encouraged to spend time building rapport and trust with participants prior to delivery of SFL components. Informal delivery respects the ethos of the Sheds and facilitates comfort and active participation. Moreover, trust facilitates a sense of safety and a positive dynamic where participants can be open and honest. This was also an important facilitator in promoting understanding of Shedders and Sheds for all stakeholders alongside the capacity building focus of the GEEMS and ENGAGE training.

Feasibility and cost for the POs must be viewed in the context of continually shifting variables within the wider implementation climate. For instance, while adoption and POs' commitment to SFL remain high, these organizations are predominately NGOs meaning that sustained funding can be precarious. Therefore, commitment is largely contingent on determinants such as staff capacity and funding as well as key implementers and leaders within the individual POs who maintain support and momentum for SFL. This must also be considered in terms of the capacity of POs for scale-up of SFL. While POs may be committed to scaling up, funding structures are needed to support this:

“*These [POs and organization collaborations] were mutually beneficial partnerships….these provider organizations had long terms goal of working with Sheds. I think that's become very apparent over the last couple of years the POs with us are with us from the very beginning.”* Organization stakeholder

“*Its [scale-up] funding dependent. I mean we got involved obviously with Sheds for Life as did everybody because we saw the benefit and hoped that there would be future funding for it. But unless there is - I mean we couldn't continue to deliver. There is a lot of Sheds… finding out and we want to deliver but need some donation or funds to the charity to cover our time and costs”-* PO stakeholder

These determinants therefore require ongoing monitoring through continued engagement with the POs. Furthermore, in relation to appropriateness, although currently structured as a 10-week intervention with both core and optional components, SFL was designed as a flexible, dynamic program, subject to ongoing adaptation to meet evolving needs. This means that the SFL implementation strategy also needs to remain flexible to accommodate new POs over time in response to new or evolving requirements and preferences from Shedders. Thus, the structure and partnership network of SFL will inevitably evolve and grow over time. Whilst this presents certain challenges, it can also be seen as a strength of SFL, not least in terms of its potential to remain fresh and contemporary, but also its embedment in real-world conditions where determinants are understood and can be managed. It is heavily invested in a partnership network that recognizes the value of SFL and respects the ethos of Sheds.

#### Determinants at organizational level

At the organizational level, there was general acceptability of the SFL initiative as the IMSA had an existing men's health remit which was supported by external funding of the National Health Service (HSE) and mandated by men's health policy (41). While SFL took on significant momentum, this presented challenges for the organization in terms of the capacity of its small team of staff to manage the significant level of administration work required and the complexity of multiple stakeholders at Shedder, Shed, PO and systems level

“*There was so many different multiple partners and components that it was six day a week job, sometimes more”* – Organization stakeholder

This also meant that there was pressure on the organization to fulfill other competing priorities and to secure funding to support general operations and work systems. This brought potential to conflict with the ethos of SFL and Sheds themselves and meant that leadership by SFL implementers was critical to ensure implementation effectiveness of SFL. Advocacy was required in terms of highlighting the importance of the foundational work required to implement SFL, ensuring that Shedders needs remained prioritized and the Shed environment respected. This also meant careful selection of POs (as opposed to seeking partnerships or funding from organizations that didn't have consistent ideals):

“*Constantly having to try and fight that battle that they recognize health and wellbeing being is the anchor of all things Men's Sheds. Highlighting that having a presence on the ground with them is so important and that we shouldn't be removed from that in the organization. And even when we are looking at corporate sponsors because the physical and mental health is such a key aspect of the Sheds, we really need to be careful who we work with and the messages they are out there giving about that as well. You know we could take money from various different provider organizations but are they right fit? Have the right ethos for the Sheds? It is really important to know.” –* Organization stakeholder

Capacity was therefore a core determinant of SFL feasibility and scale-up both in terms of coordination and planning of SFL as well as maintaining important networks and communication at multiple levels, particularly at ground level with Sheds. The implementation of the first phase of SFL at organizational level was largely the responsibility of the health and wellbeing manager and researcher until further funding was secured for a health administration role:

“*Notwithstanding the sheer volume of work with the Sheds…the back and forth with the provider organizations who then have to work with their own individual tutors around their timetables, providing Sheds for Life stakeholder meetings as well. Organizing funding and payments for the different provider organizations and putting out MOUs and contracts with the provider organizations. It's all, all very admin intense. It certainly makes it easier now there is a fulltime administrator there to support it.”* – Organization stakeholder

While it was important at this time for key implementers within the host organization to gain insights into the implementation of SFL across multiple levels, sustaining this momentum with limited capacity could ultimately be a barrier to the sustainability of SFL. For instance, the capacity demands required at ground level meant little attention could be awarded for advocacy at a systems level:

“*In terms of managing the development of it and the implementation meant, you know, with small staff numbers that the both of us had to get involved in a lot of on the ground stuff in terms of implementation. That's been fantastic in one sense in that it's been really able to inform and direct us in how SFL should be going and constantly evolving. At the same time there is still that advocacy piece that is still needed to place health on the systems agenda in Men's Sheds that sometimes had to kind of get pushed to the side because there was so much hands on stuff.” –* Organization stakeholder

Moreover, the researcher's contribution to implementation efforts ended once the evaluation was complete. Alongside this, staff turnover is an inevitable feature of NGOs because of more limited prospects of promotion, job security and salary increments. This meant that there was limited capacity to retain staff who understood the intricacies of SFL, as well as a loss of leadership at organizational level which was also a consequence of contextual shifts due to the COVID-19 pandemic. Therefore, persistent barriers to sustainability and subsequent scale-up of SFL at organizational level are leadership and staff resources:

“*I think the fact that the program has grown significantly the whole SFL umbrella over the last four years and it was very clear from the beginning there would need to be more staff in order to upscale it and further develop it. So trying to make the case that, that you can't run a national program with one person was something that was challenging*” – Organization stakeholder

Nevertheless, the evaluation of SFL which demonstrated that the program is cost effective (35), reaches HTR groups (46) and provides important benefits in terms of health and wellbeing for Shedders (31), meant that it was possible to leverage financial support for SFL at a systems level. This meant that the Irish Men's Sheds Association was awarded ongoing funding for delivery costs of SFL under “Sláintecare”- a framework for health service reform in Ireland which focuses on preventative strategies within the community setting (47) which was integrated into a sustainable funding model under the public health framework, Healthy Ireland (42). While this funding increases the sustainability of SFL, in terms of scalability, the organization will likely need further funding support to increase capacity of staff to oversee delivery of SFL in multiple locations. While there are capacity issues that may impact scalability of SFL, the initiative has demonstrated it is an effective, transferable model that is scalable with the right leadership and support at organizational level:

“*The biggest threat, the main thing is the finances. The demand is there in the Sheds. There is enough interest from the provider organizations. The provider organizations can match out demand for delivery as long as we can give some financial contribution to it.”* – Organization stakeholder

As with all NGOs, there was significant disruption to the organization during the COVID-19 pandemic (48). Alongside staff turnover mentioned above, this impact was felt across multiple levels in terms of Shed closures and the direct impact on Shedders (49), funding disruptions and pressure to fulfill previous mandates agreed pre-pandemic. This ultimately rendered it unfeasible to deliver SFL throughout the pandemic due to multiple contextual factors beyond safety concerns, such as Shed readiness and capacity of POs to deliver. However, with the arrival of a sustainable funding stream and the evidence to support the efficacy of SFL with envisioned adaptations and leadership—the demand for SFL is likely to be high at Shedder level. While POs remain committed to SFL it will be important for the organization to continue its engagement of key stakeholders involved in SFL delivery to regain momentum and renew vigor that may have been lost during COVID-19 as well as establish new relationships required to respond to Shedders' needs post-pandemic, where the pandemic may have elevated wellbeing as a priority:

“*I think that people have this opinion that ‘Oh wellbeing is something nice and fluffy there' but the reality is that wellbeing is the difference between us being able to get up in the morning and not so it shouldn't be seen as a nice fluffy add on. It is something that should really be prioritized…being able to offer something like this to the men is being able to keep them well enough to continue to attend and return to their Sheds.”* – Organization stakeholder

#### Determinants at systems level

Operations at systems level have an important influence on the sustainability and scalability of SFL. Local communities are supportive of Sheds which is an important facilitator to implementation of SFL in terms of accessing resources at community level. While Sheds are viewed as important spaces at local level and recognized as an effective way of reaching men, there are issues with local services seeking to implement health initiatives in Sheds while operating in silo from the national organization. This could be a potential barrier to the wider acceptability of SFL if it becomes associated at Shed level with other initiatives that did not give the same level of due consideration to the need to adopt gendered approaches to program delivery, relationship building, and respecting the ethos of Sheds:

“*I suppose one of the other concerns I have is that there's other agents of the state, either in the health service or otherwise, doing work in Men's Sheds and developing their own programs, trying to get funding for them” –* HSE stakeholder

The funding of NGOs is also an important systems level determinant of sustainability of SFL. While NGOs are important contributors to preventative services, funding is a prevailing issue which has a significant impact in their capacity to deliver as well as recruit and retain important staff members that are often overworked and under rewarded (50). This was amplified during the COVID-19 pandemic and is an important variable in the implementation of SFL in terms of both the overseeing body and the POs capacity to deliver.

The strength and quality of evidence gathered was a key determinant of acceptability and adoption of SFL at a systems level. Policy, research and practice work also supported the need for men's health initiatives at community level (41) which were further incorporated into strategic frameworks at policy level (42). Furthermore, as previously highlighted, the evidence from the SFL evaluation helped in securing funding under Slaintecare –(47). This was fortuitous for SFL as the program fit the remit of Slaintecare reform and also the new “Healthy Communities” health service structures which focus on addressing health inequalities through a geographical (area-based) population profiling and segmentation approach (47). This approach has the potential to place SFL on a more solid footing within the implementation system without betraying the essence or integrity of the program.

### Scale up of Sheds for life

**Figure 4:**
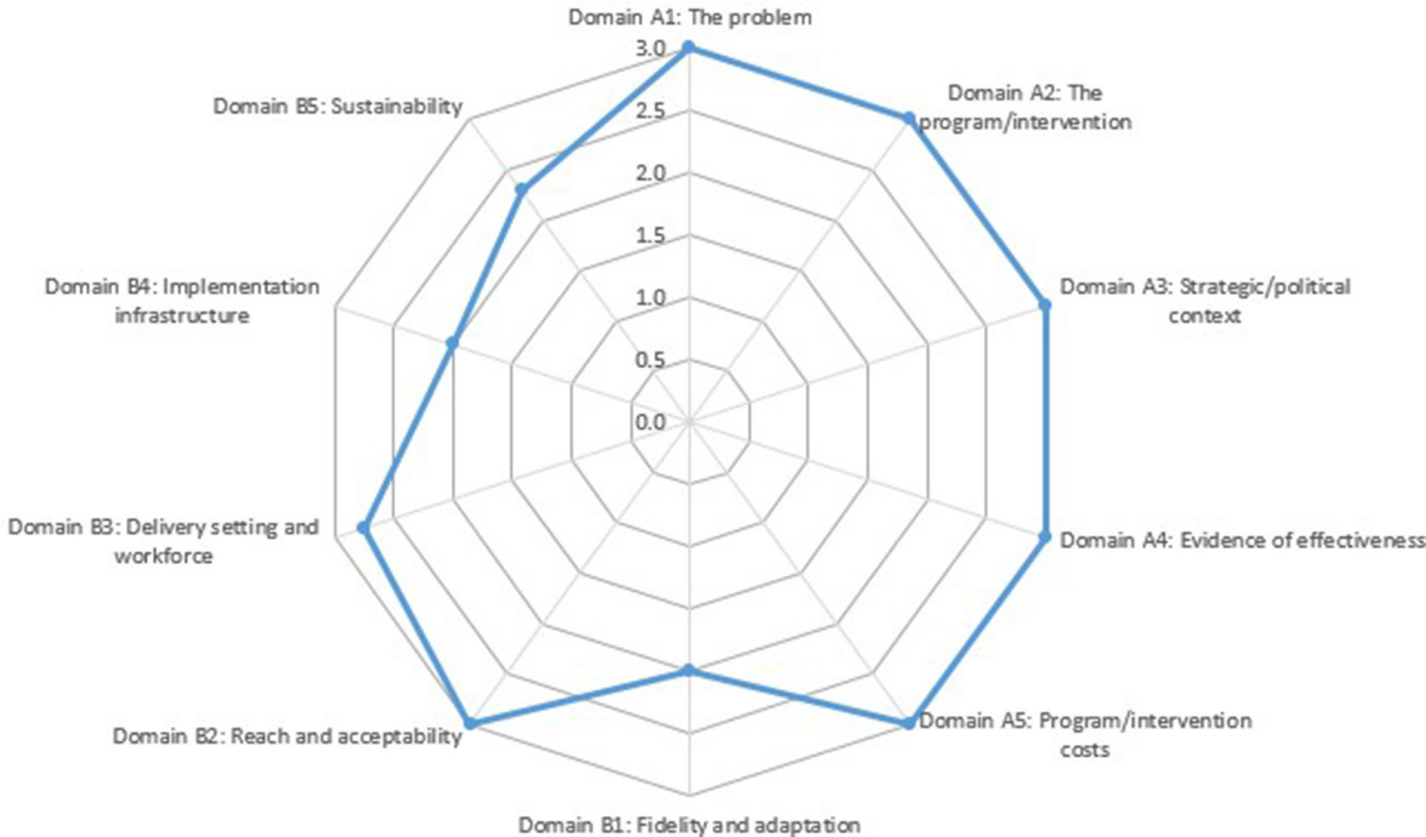
Sheds for life scalability assessment using ISAT (30). ISAT, intervention scalability assessment tool.

Finally, when scoring the readiness of SFL scalability using the Intervention Scalability Assessment Tool (ISAT) (30), SFL is an initiative that merits scale-up, providing careful attention is paid to fidelity, workforce capacity and leadership (see Figure 3). Assessment of scalability has determined a horizontal scale-up approach as most suitable within the SFL context (30). This is defined as the introduction of SFL across different Shed settings in a phased manner following the pilot through a stepwise expansion, learning lessons along the way to help refine further expansion (30). The SFL assessment highlights several domains (particularly across part A) that are high scoring while other domains scored lower as outlined in Figure 4. For further insights into the scoring of SFL scalability see Supplementary File 1.

## Discussion

This research describes the process and determinants of SFL implementation both of which inform implementation effect. The careful selection of implementation frameworks was an important facilitator toward guiding this work which helped to limit further complexities of an already complex implementation climate (36, 51). For the SFL evaluation, three frameworks were applied to guide the process, identify determinants and capture outcomes (24, 32, 33) which proved important for the research team when applying a new and innovative science to evaluation.

This work has highlighted the value of implementation research in monitoring the complexities of a multi-level co-developed intervention. While participatory research approaches are critical to the success of complex real-world innovations such as SFL they require a long-term process with commitment to sustainability (26). This can present challenges when attempting to reconcile limited community-based resources with what is needed to capture the complexities of implementation within a system of continually shifting variables (52). Indeed, a limitation to this research is the capacity of a small research team to monitor all levels of implementation and therefore it is possible that important determinants at either Shedder, PO, organizational or systems levels may have been overlooked. In the case of SFL however, it is important to remember that complexity is not just a property of the intervention but of the context or system into which it is placed, which includes multiple and dynamic interacting parts that generate nonlinear relationships (52). While this research may not provide a definitive list, it plays an important role in capturing the process of implementation for scale-up of SFL as well as providing a blueprint for other community-based health initiatives in general, and men's health initiatives in particular, that may stand to benefit from this process. The messiness of implementation requires strong leadership and advocacy which was a core determinant of SFL's successful implementation. Implementation science requires strong partnerships between the implementers and researchers involved in the intervention (53). For SFL this working partnership provided valuable momentum to implementation efforts. However, when the research becomes part of the implementation process, there is a risk that when this active ingredient is removed from further implementation that the effect may be impacted - a potential unintended consequence of implementation approaches (54). For instance, in the case of SFL the researcher spent hundreds of hours within Sheds discussing SFL and engaging with and building relationships with Shedders. Indeed, Shedders may not have distinguished the evaluation from the intervention. The researcher was placed at the epicenter of a small, albeit national organization, which oversaw the implementation and assumed multiple roles within the implementation efforts, particularly during COVID-19. This means that the researcher becomes a core part of implementation efforts. In this case the researcher was not solely viewed as external consultant but rather a key advisor within the SFL team (55). Understandably, this can raise questions about objectivity and impartiality which required the researcher to navigate ethical implications of an implementer/researcher role. Indeed this work mirrored many of the first-hand experiences captured by Cheetham, Wiseman (56) of how researchers can be subject to political pushes, pressures and sense of accountability. However, the assistance of the research team, SFL advisory, consultation with international academics and local researchers, combined with an open and transparent process of knowledge co-production with SFL stakeholders along with assertive boundary negotiations, were important in facilitating the embedded researcher to remain independent and impartial. Embedding a researcher has advantages too (55, 56), particularly in the case of public health and community-based organizations which may not have the resources to conduct rigorous evaluation, where funding is short-term and staff are heavily involved with hands-on activities. Indeed, Wolfenden et al. (55) argue that the challenges and costs of evaluating intervention trials, particularly those assessing the impact of implementation strategies, means that trials testing the impact of health interventions or implementation strategies represent 11 and 2% of research output, respectively. This research therefore provides a valuable contribution to translational research and, in terms of the sustainability of SFL, the dissemination of the findings is proving valuable in leveraging further resources. Nevertheless, understanding the role of researchers at the intersection of academia and community-based practice is an important consideration for implementation science efforts.

While this research has highlighted multiple determinants that impacted and continue to impact SFL implementation, effective strategies outlined such as the gender-specific approaches at Shed level have increased the potential for, and demonstrated the utility of, the Shed setting as a suitable environment for SFL implementation. It has demonstrated that the model is transferrable despite the variability of Sheds when determinants such as the importance of relationship building, active recruitment and co-design processes are considered. An important question for SFL is ultimately what fidelity of the initiative looks like, particularly post pandemic. Indeed, while SFL is currently structured as 10-week intervention with multiple program offerings, this implementation science study highlights that while there should be fidelity to core components of SFL in terms of content to retain effect (31), the process of implementation and key implementation strategies are perhaps more critical to SFL fidelity than strict adherence to program content. In fact the inherent nature of Sheds means a constantly changing practice environment which is a key challenge for implementation research (51). A critical juncture for SFL scalability, to potentially 450 Sheds in Ireland, will be its ability to maintain the co-designed nature of SFL, and the time spent investing in relationships with Sheds. In fact, without Shedders' acceptability and perceived appropriateness of SFL, there will ultimately be no implementation as the Sheds rightfully own SFL. The importance of these approaches is highlighted in the wider context of men's health research where the focus on addressing gender inequality in health programming has become more clearly conceptualized as a gender-transformative approach (57).

Considering the Milat et al. (29) guide to scaling up health interventions, the Sheds for Life evaluation has met the criteria of assessing effectiveness, reach, adoption, its ability to align with the strategic context, and acceptability and feasibility have been demonstrated. Moreover, scoring of the ISAT tool has showcased the initiative to meet the criteria for scale-up with careful attention required for fidelity, leadership and capacity (30). Furthermore, Indig et al. (58), discuss how interventions found effective in a controlled setting should be scaled-up and an added strength to SFL is its implementation testing in what was certainly an uncontrolled and unpredictable environment. However, scale-up is a complex process and applying a multi-level perspective on transition to scale is required (59). Moreover, while SFL has had a demonstrable impact on the health and wellbeing outcomes of Shedders, dilution of this impact should be avoided and often in the process of scaling-up health interventions, the effectiveness is reduced due to difficulties in maintaining the dose and fidelity of the original implementation (29).

This research has determined that currently SFL is an appropriate and acceptable model that has been widely adopted at Shed and PO level, while also establishing itself as a leading priority program for its host organization. The hybrid-effectiveness design of the SFL evaluation has demonstrated that SFL has emerged as the most appropriate model to reach the target cohort of HTR men (12). Moreover, it has captured the implementation process and identified important facilitators and barriers to enhance implementation efforts. It is also efficacious (31) and cost-effective (35). It is a scalable model that has also now established itself within the systems environment. The future of SFL and its potential to continue to engage Shedders and enhance their health and wellbeing outcomes is bright. Its scalability largely relies on leadership, financial and human resources and increased capacity for staff to oversee its delivery. Scaling up using a horizontal scale-up approach which introduces SFL to Sheds in a phased manner is feasible and yet requires continued refinement during further expansion (30). This approach by its very definition means it is important that research efforts remain to monitor the scalability of SFL in order for the initiative to retain fidelity to its ethos and integrity as it begins to scale up nationally. Indeed, real-word implementation means that, even if it were possible to ensure that all implementation barriers to scalability were identified and subsequently addressed, additional threats to the implementation and scale-up process that are not anticipated will likely emerge (60). Milat et al. (29), in a guide to scaling up interventions, place emphasis on subsequent evaluation and monitoring efforts during scale-up that focus on measuring effectiveness over time as well as other important implementation outcomes such as levels of penetration, adoption and acceptability. Nevertheless, our identification of implementation strategies (Table 3) provides tangible examples for researchers and practitioners that can act as a “how to” guide for successful implementation of community-based interventions. The key determinants highlighted in this work demonstrate that understanding the influence of the *process* is as important as the outcome. While effectively guiding the process can be complex, this can be made more manageable by using the right implementation approach. The implementation process must recognize the value of investing time in relationships and capacity building through working in partnership. This is the very essence of community-based work and can mean the “how” of implementation is as health enhancing as the “what”'.

## Conclusion

This research has captured the process and determinants of effective implementation of a community-based men's health promotion programme. Guided by implementation science, it has informed the scalability of SFL as well as identifying a “how to” of implementation strategies that can act as a blue print for other men's health settings and programs and health promotion more broadly. The evaluation of SFL highlights the importance of knowledge co-production in men's health work as well as in translational and implementation research efforts. While the evaluation of real-world multi-level interventions is complex, this work highlights the value and utility of embedded research which facilitates iterative decision making and allows adaptions to implementation subsequently promoting translation of research and knowledge production into practice in real-time. The evaluation demonstrates the importance of gender-specific approaches to men's health promotion where co-designed processes can help to positively redefine what health engagement means to HTR men. This work highlights that the process of implementation is as critical as the content that is delivered, meaning fidelity to the process is fundamental to retain effectiveness in scale-up efforts. This is the first evaluation to capture an implementation process of health promotion in Sheds. Moreover, this work makes a valuable contribution to research where there exists a dearth of research outputs capturing implementation strategies. It offers practitioners and researchers an example of the operationalization of implementation frameworks in practice as well as identifying strategies to engage key stakeholders, the most important of which are those who will ultimately use, and should rightfully own, the intervention. Therefore, real-world interventions should be designed with this in mind through strengths based, grassroots approaches.

## Data availability statement

The raw data supporting the conclusions of this article will be made available by the authors, without undue reservation.

## Ethics statement

The studies involving human participants were reviewed and approved by Waterford Institute of Technology Research Ethics Committee (REF: WIT2018REC0010). The patients/participants provided their written informed consent to participate in this study. Written informed consent was obtained from the individual(s) for the publication of any potentially identifiable images or data included in this article.

## Author contributions

AM, NM, and NR: conceptualization, methodology, funding acquisition, project administration, visualization, and writing—review and editing. AM: investigation and writing—original draft. NM and NR: supervision. All authors have read and agreed to the published version of the manuscript.

## Funding

AM was supported through an Irish Research Council Doctoral Award (Project ID EBPPG/2018/256). The funders were not involved in the design of the study, manuscript writing or collection of data, nor were the funders involved in data analysis or in manuscript writing.

## Conflict of interest

The authors declare that the research was conducted in the absence of any commercial or financial relationships that could be construed as a potential conflict of interest.

## Publisher's note

All claims expressed in this article are solely those of the authors and do not necessarily represent those of their affiliated organizations, or those of the publisher, the editors and the reviewers. Any product that may be evaluated in this article, or claim that may be made by its manufacturer, is not guaranteed or endorsed by the publisher.

## Abbreviations

CBPR: Community-based participatory research
SFL: Sheds for Life
IMSA: Irish Men's Sheds Association
HTR: Hard-to-Reach
CFIR: Consolidated Framework for Implementation Research
PO's: Provider organizations
PRACTIS: Practical Planning for Implementation and Scale-up guide
GEEMS: Guidance for Effective Engagement with Men's Sheds.
