# Supplementary material for: Strategies for effective implementation and scale-up of a multi-level co-designed men's health initiative “Sheds for Life” in Irish Men's Sheds
Source: Front Health Serv. 2022 Nov 3;2:940031. doi: 10.3389/frhs.2022.940031 (PMC10012692; doi:10.3389/frhs.2022.940031)
Supplement: Supplementary file 1 [file Table_1.DOCX]

| **Supplementary File 1: ISAT scoring for horizontal scale up* of Sheds for Life** | | | | | |
| --- | --- | --- | --- | --- | --- |
| ***Domain A1: The Problem*** | | | | | |
| Is the problem of sufficient concern to warrant scale up of the intervention/program to address it? | N/A | 0 | 1 | 2 | 3 |
| ***Domain A2: The intervention*** | | | | | |
| Will the outcomes delivered by this intervention address the needs of the target group (and/or) problem? | N/A | 0 | 1 | 2 | 3 |
| ***Domain A3: Strategic/Political Context*** | | | | | |
| Is addressing the problem consistent with policy/strategic directions or priorities? | N/A | 0 | 1 | 2 | 3 |
| Will scaling up the intervention be strategically useful to funders/funding agency? | N/A | 0 | 1 | 2 | 3 |
| Average score for Domain A3 | 3 | | | | |
| ***Domain A4: Evidence of Effectiveness*** |  |  |  |  |  |
| Is there compelling evidence (from the literature or elsewhere) to indicate that the intervention is effective in addressing the problem in the target population? | N/A | 0 | 1 | 2 | 3 |
| ***Domain A5: Intervention costs and benefits*** | | | | | |
| Is there evidence that the benefits of the intervention exceeded the costs? | N/A | 0 | 1 | 2 | 3 |
| ***Domain B1: Fidelity and adaptation*** | | | | | |
| Will the core components of the scaled up intervention be consistent with what was previously shown to be effective? | N/A | 0 | 1 | 2 | 3 |
| If the core components of intervention are to be changed/adapted from its original form during scale up, will the impact of the changes/adaptations likely be favourable? | N/A | 0 | 1 | 2 | 3 |
| Can program fidelity be monitored and/or maintained if implemented at scale? | N/A | 0 | 1 | 2 | 3 |
| Average score for Domain B1 | 2 | | | | |
| ***Domain B2: Reach and acceptability*** | | | | | |
| Does the intervention have the potential to reach the intended target population at scale? | N/A | 0 | 1 | 2 | 3 |
| Is the intervention likely to be acceptable to the target population? | N/A | 0 | 1 | 2 | 3 |
| Average score for Doman B2 | 3 | | | | |
| ***Domain B3: Delivery setting and workforce*** |  |  |  |  |  |
| Is the delivery setting(s) selected to deliver the program at scale consistent with that used in previous studies? | N/A | 0 | 1 | 2 | 3 |
| Is the delivery workforce selected to deliver the program at scale consistent with that used in previous studies? | N/A | 0 | 1 | 2 | 3 |
| Is the intervention likely to be acceptable to the delivery workforce involved in its delivery at scale? | N/A | 0 | 1 | 2 | 3 |
| If the intervention requires integration into existing organisational or community structures, how likely is it to be feasible? | N/A | 0 | 1 | 2 | 3 |
| Average score for Domain B3 | 2.75 | | | | |
| ***Domain B4: Implementation infrastructure*** | | | | | |
| Are the implementation infrastructure requirements of the intervention/program feasible for scale up**?** | N/A | 0 | 1 | 2 | 3 |
| ***Domain B5: Sustainability*** |  |  |  |  |  |
| *Is the level of integration of the intervention into delivery settings required for implementation at scale sustainable?* | N/A | 0 | 1 | 2 | 3 |
| *Is the level of resourcing required to implement the intervention at scale sustainable?* | N/A | 0 | 1 | 2 | 3 |
| *Is the delivery workforce selected for implementation at scale sustainable?* | N/A | 0 | 1 | 2 | 3 |
| Average score for Domain B5 | 2.33 | | | | |
| *N/A = not applicable 0=Not at all 1=to a small extent 2=somewhat 3= to a large extent*  *Scoring:* The purpose of these readiness questions is to assist in identifying the strengths and weaknesses across the domains. Each question is scored from 0–3, where the minimum score for each domain is 0 and the maximum score is 3. In order to derive a final score for the domain, the average score across the questions is taken (if there is more than one question).  ** Scaling up using a horizontal approach involves the introduction of an intervention across different sites or groups in a phased manner, often beginning with a pilot program, followed by stepwise expansion, learning lessons along the way to help refine further expansion* (Milat et al., 2020) | | | | | |
